# Primary Hyperparathyroidism

**DOI:** 10.12688/f1000research.7039.1

**Published:** 2016-01-04

**Authors:** Leonardo Bandeira, John Bilezikian

**Affiliations:** 1 Department of Medicine, College of Physicians and Surgeons, Columbia University, New York, NY, USA

**Keywords:** Primary Hyperparathyroidism, parathyroid glands, adenomas

## Abstract

Over the past several generations, primary hyperparathyroidism (PHTP) has undergone a change in its clinical presentation in many countries from a symptomatic disease to an asymptomatic one. The reasons for this change in clinical presentation are related to the widespread use of biochemical screening tests, to the measurement of PTH more routinely in the evaluation of metabolic bone disease and to the status of vitamin D sufficiency in the population. Along with recognition of a broader clinical spectrum of disease, including a more recently recognized normocalcemic variant, has come an appreciation that the evaluation of classic target organs that can be affected in PHPT, such as the skeleton and the kidneys, require more advanced imaging technology for complete evaluation. It is clear that even in asymptomatic patients, evidence for microstructural disease in the skeleton and calcifications in the kidneys can be demonstrated often. Potential non-classical manifestations of PHPT related to neurocognition and the cardiovascular system continue to be of interest. As a result of these advances, revised guidelines for the management of asymptomatic PHPT have been recently published to help the clinician determine whether surgery is appropriate or whether a more conservative approach is acceptable.

## Introduction

Primary hyperparathyroidism (PHPT) is characterized by hyperactivity of one or more parathyroid glands, disordered calcium homeostasis, and a consequent increase in serum calcium and elevated or inappropriately present circulating levels of parathyroid hormone (PTH) ^1^. In the 1940’s Fuller Albright described the classic manifestations of PHPT as a disorder of bones and stones ^2^. Indeed, historically, PHPT was characterized best by skeletal and kidney involvement. This clinical landscape changed rather dramatically in the 1970s with the advent of the multichannel autoanalyzer that provided a serum calcium concentration whenever the biochemical screening test was ordered. The diagnosis of PHPT was made much more commonly thereafter by mild hypercalcemia, lack of any specific symptomatology, or obvious renal or bone disease ^3^. Currently, up to 80% of patients with PHPT in parts of the world where biochemical screening is routine have “asymptomatic” PHPT ^1^.

Most patients with PHPT have a single, benign adenoma. A smaller percentage, about 15% to 20%, have multigland disease, including multiple adenomas and hyperplasia. Multigland disease is more common in familial syndromes such as multiple endocrine neoplasia (MEN) 1 or 2 ^4^. Parathyroid carcinoma is rare, occurring in fewer than 1% of patients with PHPT ^1^.

Gene mutations can be associated with the development of parathyroid tumors. These genes include MEN1, calcium-sensing receptor (CASR), HRPT2, RET (familial forms), and PRAD1/cyclin1 (sporadic tumors) ^1, 5^.

## Epidemiology

PHPT is the leading cause of hypercalcemia ^6^ and one of the most common endocrine disorders; the estimated prevalence is between 0.1% and 0.5% in the US ^3, 7^. It is observed mainly in postmenopausal women (female-to-male ratio of 3–4:1) over 50 years old ^6, 8^. A large Brazilian study showed a prevalence of 0.78%, and the vast majority of these patients were asymptomatic (81%) ^7^.

In younger patients, PHPT should call attention to the possibility of MEN, especially type 1, because this genetic syndrome has rather high penetrance in the decades up to 30 years of age. By age 50, hypercalcemia will have surfaced in virtually all patients with MEN-1. Besides its earlier onset, PHPT in MEN-1 has a gender distribution more likely to be balanced and bone and renal involvement is likely to be more evident ^4^. Autopsy studies have estimated the prevalence of MEN-1 in PHPT to be 1% to 18% ^9^. A multicenter study conducted in Italy, involving 533 patients with PHPT, found a MEN-1 prevalence of about 13%. This study also showed that patients with MEN-1 were younger ^10^.

## Diagnosis

The diagnosis of PHPT is very straightforward after confirmation of persistent hypercalcemia. The associated PTH level is frankly elevated or inappropriately normal. In the latter regard, PTH levels can be as low as the “20s” (with a normal range of 10 to 65 pg/mL) and still be regarded as inappropriate for a hypercalcemia state ^11^. The total serum calcium must be adjusted for albumin. For every gram-per-deciliter reduction in the serum albumin concentration, the total calcium measurement should be adjusted upwards by 0.8 mg/dL. In regard to PTH, either the second generation (measuring the intact molecule and large inactive fragments) or third generation (measuring the intact PTH molecule exclusively) can be used for diagnosis ^1^.

Another form of PHPT has been recognized over the past 15 years. Normocalcemic PHPT (NPHPT) is characterized by elevated PTH levels in the presence of serum and ionized calcium that are consistently normal. Secondary causes of an elevated PTH—such as renal compromise (creatinine clearance of less than 60 mL/min), 25-hydroxyvitamin D insufficiency (less than 30 ng/mL or 80 nmol/L), primary hypercalciuria, malabsorption syndromes, and medications such as lithium or thiazides—must be excluded. In patients who present with normal levels of serum calcium, high PTH concentrations, and vitamin D deficiency, treatment with vitamin D can lead to the common hypercalcemic form of PHPT ^3^.

Also important in the evaluation of PHPT is a 24-hour urine collection. A calcium clearance-to-creatinine clearance ratio (CaCrR) will help in differentiating familial hypocalciuric hypercalcemia (FHH), a rare disorder that can be confused with PHPT. FHH is an autosomal dominant disease characterized by mild hypercalcemia, very low urine calcium excretion, and elevated serum PTH. It is caused by a mutation in the CASR gene. It is important to distinguish FHH from PHPT because FHH is a benign condition for which parathyroid surgery is virtually never indicated ^12^. A CaCrR of less than 0.01, on a normal dietary calcium intake, is observed in 80% of patients with FHH. There is some overlap between FHH and PHPT, particularly in the CaCrR range of 0.01 to 0.02. However, it is unlikely in FHH for the CaCrR to exceed 0.02. Similar to the MEN syndromes, FHH displays high genetic penetrance and hypercalcemia becomes evident usually before the age of 30. There is also often a family history of hypercalcemia ^12, 13^.

In addition to the determination of urinary calcium excretion in PHPT, it is important to evaluate other urinary constituents that together may comprise stone risk in these patients. Whereas urinary calcium excretion alone is not helpful vis-à-vis determination of stone risk in PHPT, the complete urinary biochemical stone risk profile is. Therefore, the 24-hour urine collection in PHPT should include a full urinary stone biochemical risk profile ^14^.

## Clinical presentation

PHPT presentations vary worldwide. This is particularly true now with the availability of the autoanalyzer that varies in usage throughout the world. Where biochemical screening is routinely performed, PHPT usually presents with mild hypercalcemia. Overt bone and stone diseases are unusual ^15^. A good illustration of how the availability of biochemical screening can influence the clinical presentation of PHPT is seen in the study comparing PHPT in the US and China. In a cohort studied in New York City, 80% of subjects were asymptomatic, whereas virtually everyone in Beijing was symptomatic ^16^. Thirteen years later, in 2013, China showed a reduction in symptomatic PHPT to 60% and this was due presumably to the wider availability of multichannel biochemical screening in that country. In the US, the disease continues to present primarily asymptomatically (80% of patients). However, a new phenotype, NPHPT, has emerged (see below) ^17^.

Although the trend for asymptomatic PHPT to become progressively more the dominant form of PHPT is clear worldwide, symptomatic PHPT continues to be the predominant form of the disease in some countries, such as India, Iran, and Saudi Arabia ^15^.

In Brazil, an increase in the incidence of asymptomatic PHPT has also been observed recently. Within a decade, albeit in different cities, the incidence of asymptomatic PHPT increased from 34% in São Paulo to 82% in Recife ^7, 8^. Data from other Latin American countries are limited, but it seems that the majority of patients still present with symptomatic PHPT ^15^.

### Symptomatic disease

Osteitis fibrosa cystica (OFC) is the classic presentation of skeletal involvement in PHPT. Characteristic radiological features include a salt-and-pepper appearance in the skull, phalangeal subperiosteal bone resorption, cysts, and brown tumors. In addition, bone pain, skeletal deformities, and pathological fractures can be present ^18^. Typically, bone mineral density (BMD) is very low and bone markers are elevated. Radiologically and densitometrically, cortical bone is more involved than trabecular bone. Thus, skeletal sites with a predominance of cortical bone, such as the distal third radius, will show greater bone loss. By dual-energy X-ray absorptiometry (DXA), the distal 1/3 radius site, thus, is preferentially affected ^14^.

Nephrocalcinosis and nephrolithiasis are the most common complications of PHPT ^14^. In Shanghai, where approximately 60% of the patients are symptomatic, the prevalence of kidney stones among patients with PHPT is 48% ^17^. Hypercalciuria contributes to the formation of stones but the etiology is multifactorial ^14^.

PHPT can affect organs other than the skeleton and kidney when it presents as a symptomatic disease. Other affected organ systems include the neuromuscular (peripheral polyneuropathy and proximal muscle weakness), gastrointestinal tract (peptic ulcer and pancreatitis), cardiovascular (hypertension, atherosclerosis, left ventricular hypertrophy, valve calcification, and arrhythmias), and psychiatric (anxiety, irritability, apathy, psychosis, and loss of memory) disorders ^15, 18, 19^. Also, manifestations related to symptomatic hypercalcemia can be present; these include nausea, vomiting, constipation, polyuria, polydipsia, mental confusion, coma, and a short QT interval on the electrocardiogram ^20^. It should be emphasized that multi-organ involvement in PHPT is the classic presentation of symptomatic disease. The extent to which these organ systems may be involved in asymptomatic disease is not clear (see below).

### Asymptomatic disease

Owing to the prevalence of asymptomatic PHPT, OFC is rarely seen these days in developed countries. However, skeletal involvement continues to be readily detected by technologies such as DXA ^15, 21^. Reduced BMD is observed most often in distal 1/3 radius site, comprised mostly of cortical bone, with relative preservation of lumbar spine, a site that is endowed primarily with trabecular bone ^22^. However, these densitometric findings are not consistent with epidemiological data that have consistently reported a greater incidence of fractures at trabecular (vertebral) as well as cortical (non-vertebral) sites in PHPT. Vignali *et al.* evaluated the incidence of vertebral fractures (VFs) in postmenopausal women with PHPT as compared with a control group. Patients with PHPT, regardless of whether they were symptomatic, had more VFs (24.6% versus 4.0%, *P* <0.0001) ^23^. Also, there was no difference in VF risk between symptomatic and asymptomatic patients (34.1% versus 21.1%, *P* = 0.15) but compared with matched non-hyperparathyroid control subjects, these figures were clearly and significantly higher ^23^.

Another study published recently compared the incidence of VF in patients with symptomatic and asymptomatic disease. Both groups had a high percentage of VFs (34.4% and 34.7%) but the difference between the two groups was not appreciated ^24^. The availability of new technologies such as high-resolution peripheral quantitative computed tomography (HRpQCT) and trabecular bone score (TBS) has provided a greater understanding of skeletal microstructure in asymptomatic patients ^22^.

HRpQCT analyzes images from peripheral sites, such as the radius and tibia, and is capable of discerning microstructural characteristics. It can discern trabecular and cortical compartments separately with regard to volumetric bone density, bone geometry, cortical thickness, and trabeculae distribution and number ^25^. A recent study showed, by HRpQCT, that patients with PHPT versus controls had decreased volumetric densities at trabecular and cortical compartments, thinner cortices, and more widely spaced and heterogeneously distributed trabeculae. These changes were more pronounced at the radius, suggesting a protective effect of increased mechanical load on the tibia, a weight-bearing site ^26^. A study with a similar design found changes at cortical and trabecular bone in patients with PHPT as compared with a control group but only at the radius and not on the tibia ^27^.

TBS is a textural index that evaluates pixel gray-level variations in the lumbar spine DXA image, providing an indirect index of trabecular microarchitecture. It can be readily applied to a DXA image through the use of a specific software program that is approved by the US Food and Drug Administration. A low TBS value is associated with fewer, less well-connected, and more widely distributed trabeculae ^22, 28, 29^. Studies have shown a positive correlation between TBS values and HRpQCT indices ^30, 31^. Silva *et al.* evaluated TBS, compared with HRpQCT findings in 22 postmenopausal women with PHPT, most of whom were asymptomatic ^31^. TBS revealed a degraded trabecular index of 1.24 (normal is more than 1.35). Moreover, the TBS value was correlated with many HRpQCT indices of trabecular microarchitecture.

These new imaging technologies have revealed abnormalities in trabecular bone in addition to the well-known cortical involvement in PHPT. They help to resolve the clinical paradox of relatively normal lumbar spine BMD by DXA in the context of an increase in VFs in PHPT ^21, 22^.

The incidence of nephrolithiasis in patients with PHPT has declined as the percentage of patients with asymptomatic PHPT has increased ^21^. However, when imaging studies are conducted among subjects who have no history or symptoms of renal stone disease, nephrolithiasis or nephrocalcinosis (or both) can be detected rather often ^32, 33^.

Non-traditional manifestations of PHPT with particular regard to cardiovascular and neurocognitive features are the subject of interesting but inconclusive studies. At this point, we cannot reach any conclusions about either of these “off-target” systems with regard to their direct relationship to PHPT (see neurocognitive features in the “Quality of life” section). Although cardiovascular morbidity and mortality seem to be increased in patients with severe PHPT, these findings have not been confirmed in those with asymptomatic disease. Among these complications, increased vascular stiffness is the one that has been most consistently demonstrated. Other findings, such as cardiovascular mortality, hypertension, coronary artery disease, valvular calcification, ventricular hypertrophy, arrhythmias, diastolic dysfunction, and carotid atherosclerosis, are characterized by limited and conflicting data in asymptomatic patients ^34^.

### Normocalcemic disease

Most reports on NPHPT come from referral centers in which subjects were evaluated for an osteometabolic disease ^35^. Lowe *et al.* evaluated 37 patients with NPHPT and found rather high prevalence rates of osteoporosis (57%), fragility fractures (11%), and nephrolithiasis (14%) ^36^. Amaral *et al.* compared patients with NPHPT and mild hypercalcemic PHPT and found no difference in prevalence of fractures and nephrolithiasis between the groups ^37^. Charopoulos *et al.* examined the effects of PHPT in the skeleton by using peripheral quantitative CT and noted that the trabecular compartment is preserved in NPHPT ^38^. In studies that have indicated more skeletal involvement than one might expect in NPHPT, the selection bias from a metabolic bone diseases center might be relevant.

Data are conflicting and limited about the natural history of NPHPT. Lowe *et al.* observed disease progression in 40% of patients ^36^. However, other studies, with subjects randomly assigned from community populations, showed no progression to overt PHPT ^39, 40^. The prevalence of NPHPT varies widely among different reports and this is probably due to methodological differences. For example, two studies published in 2015, both performed with randomly assigned community populations in Sweden and Italy, found a prevalence of 11% and 0.44%, respectively, when using different cutoff values for PTH and 25-hydroxyvitamin D ^40, 41^.

We cannot draw many conclusions at this stage of our understanding of NPHPT, but it is fair to state that the absence of hypercalcemia does not imply that NPHPT is a mild or an asymptomatic form of PHPT. A significant proportion of patients with NPHPT can show characteristics similar to those of patients with symptomatic or asymptomatic hypercalcemic PHPT ^22^. Screening of an unselected population can unmask subjects with NPHPT with no apparent signs or symptoms ^42^. Thus, as is the case for the more common hypercalcemic variant of the disease, patients with NPHPT can present a spectrum of target organ involvement. Also, similar to its more established hypercalcemic counterpart, NPHPT has a variable natural history without any clear-cut factors that can predict who will or will not show disease progression ^35^.

### Quality of life

The “moans and groans” of PHPT, as classically described by Albright in the 1940’s, haunt us today because so many patients complain of non-specific neurocognitive issues. With the change in clinical presentation of PHPT, these neurocognitive issues have surfaced only to confound rather than to clarify a possible association between them and the disease. A central question, thus, relates to whether PHPT is associated in a causative way with these complaints. Some studies have found this association, although it remains unclear how much the events can be ascribed specifically to PHPT. Caillard *et al.*, in a multicenter prospective study, analyzed patients with PHPT before and after a successful parathyroidectomy ^43^. Preoperatively, non-specific symptoms were common: anxiety, body pain, abdominal distention, forgetfulness, headaches, and mood swings. Significant improvement in quality of life was found 3, 6, and 12 months after surgery. Veras *et al.* compared a group of patients with newly diagnosed PHPT with another group of patients with more long-standing disease and found that the latter were affected more in functional capacity, physical limitation, general health, and vitality ^44^. Amstrup *et al.* found poor quality of life in patients with PHPT compared with a control group, even after parathyroidectomy ^45^. These studies, using a generic quality-of-life questionnaire SF-36 (36-Item Short Form Health Survey), are by no means conclusive ^43– 45^.

In a randomized controlled trial with only asymptomatic patients, Rao *et al.* used scores to measure quality of life and psychosocial function, comparing a group that underwent parathyroidectomy and another group that was followed without surgery ^46^. There was an increase in scores for social and emotional function in addition to declines in anxiety and phobia in the group that had surgery. Walker *et al.*, in a case-control study that used several tests to measure cognitive function, found weaker performance in various domains such as depression, anxiety, and memory in patients with PHPT compared with a control population ^47^. Parathyroidectomy led to an improvement in some of the cognitive parameters. However, no linear association between calcium or PTH and these abnormalities was noted.

## Guidelines for the management of primary hyperparathyroidism

Patients with classic symptoms of PHPT should be referred for surgery ^48, 49^. The vexing question is who among those with asymptomatic disease should undergo parathyroidectomy. Four international workshops on the management of asymptomatic PHPT have addressed this question over the past 25 years ^48– 50^. The most recent one, in 2013, led to revised guidelines based upon advances over the previous 5 years. The newer observations have led the experts to recommend a more proactive approach to detect target bone and kidney involvement. The current indications for surgery in asymptomatic PHPT are as follows ( Table 1) ^48^:

1. Serum calcium value of more than 1 mg/dL above the upper limit of normal.
2. Peri- or post-menopausal women and men at least 50 years old who have a T-score of not more than −2.5 at the lumbar spine, femoral neck, total hip, or distal 1/3 radius. In premenopausal women and men younger than 50 years old, a Z-score of not more than −2.5 is recommended as the cut-point. Other approaches to skeletal evaluation (X-ray, VF assessment, TBS, and HRpQCT) are recommended to determine whether skeleton involvement is present. Substantial trabecular disease would support a decision for surgery. If a VF is present, surgery is clearly recommended.
3. Creatinine clearance of less than 60 mL/minute.
4. Presence of nephrolithiasis or nephrocalcinosis. Evaluation with imaging studies, such as X-ray, computed tomography (CT), or ultrasound, is now recommended.
5. Presence of hypercalciuria (more than 400 mg/day) along with a complete urinary biochemical stone risk profile that places the patient at risk for nephrolithiasis or nephrocalcinosis.
6. Age of less than 50 years.

**Table 1.** Current indications for surgery in asymptomatic hyperparathyroidism and comparison with previous ones.

Measurement	2002	2008	2013
Serum calcium (>upper limit of normal)	1.0 mg/dL (0.25 mmol/L)	1.0 mg/dL (0.25 mmol/L)	1.0 mg/dL (0.25 mmol/L)
Skeletal	BMD by DXA: T-score < −2.5 at any site	BMD by DXA: T-score < −2.5 at any site	A. BMD by DXA: T-score < −2.5 at lumbar spine, total hip, femoral neck, or distal 1/3 radius B. Vertebral fracture by X-ray, CT, magnetic resonance imaging, vertebral fracture assessment
Renal	A. eGFR reduced by >30% from expected B. 24-hour urine for calcium >400 mg/day (>10 mmol/day)	A. eGFR < 60 cc/minute B. 24-hour urine for calcium not recommended	A. Creatinine clearance < 60 cc/minute B. 24-hour urine for calcium >400 mg/day (>10 mmol/day) and increased stone risk by biochemical stone risk analysis C. Presence of nephrolithiasis or nephrocalcinosis by X-ray, ultrasound, or CT
Age, years	<50	<50	<50

BMD, bone mineral density; CT, computed tomography; DXA, dual-energy X-ray absorptiometry; eGFR, estimated glomerular filtration rate ^48^.

Non-classic manifestations of PHPT are not included in the guidelines for surgery because the evidence is not yet in hand ^14^.

**Figure 1.**
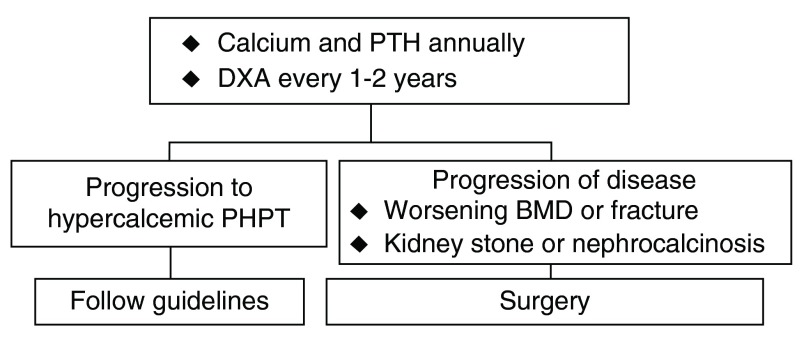
Approach in normocalcemic primary hyperparathyroidism (NPHPT) ^48^. BMD, bone mineral density; DXA, dual-energy X-ray absorptiometry; PHPT, primary hyperparathyroidism; PTH, parathyroid hormone.

Guidelines are suggesting, for the first time, an approach to NPHPT. With annual follow-up, patients who become hypercalcemic should be evaluated by the guidelines for hypercalcemic disease. If there is disease-associated progression with fractures, bone loss, nephrolithiasis, or nephrocalcinosis, surgery should be considered even if the patient continues to be normocalcemic ( Figure 1) ^48^.

Revised recommendations for monitoring patients who are not to undergo parathyroid surgery are as follows:

1. Annual serum calcium.
2. Three-site DXA every 1 to 2 years.
3. X-ray or VF assessment of spine if clinically indicated (height loss or back pain).
4. Creatinine clearance and serum creatinine annually.
5. If renal stones or other renal involvement is suspected: 24-hour biochemical stone profile plus renal imaging.

If, during follow-up, any criteria for surgery develop and there are no medical contraindications, surgery should be strongly recommended ^48^.

## Preoperative localization/surgery

All patients should undergo preoperative imaging tests before parathyroidectomy, in order to locate the affected gland(s). Imaging tests are not used for diagnostic purposes. There is, thus, no indication for parathyroid imaging if surgery is not planned. The most commonly used tests are ultrasound, sestamibi imaging, and CT. The use of CT with four-dimensional techniques (4D-CT) is providing greater anatomical resolution ^51^. A recent study that used imaging with 4D-CT only in patients whose parathyroid adenoma had not been identified with ultrasound and sestamibi showed a sensitivity of 89% ^52^. Another study compared the cost-effectiveness of imaging tests by using a hypothetical cohort and calculating sensitivity and positive predictive value of the tests. It concluded that using more than one imaging technique is more cost-effective because it decreases the likelihood of a bilateral exploration ^53^.

The traditional surgical approach is bilateral neck exploration, but “minimally invasive parathyroidectomy” (MIP) has gained acceptance as an attractive approach. MIP terminology refers to any operative approach, open or endoscopic, in which the goal is to remove, as non-invasively as possible, the abnormal gland. A small incision with local anesthesia is part of this concept. Although MIP has gained in popularity, the central point to remember is that the key to the successful parathyroidectomy is not so much successful localization of the parathyroid adenoma, as useful as that is, but rather identification of the expert parathyroid surgeon ^51^.

In general, during MIP, intraoperative measurement of PTH (IOPTH) is employed to confirm that removal of the gland rectifies the problem. Because PTH has a mean half-life, measured literally in seconds in patients with normal renal function, it will normalize within minutes of removal of the parathyroid adenoma ^51^. IOPTH improves the cure rate and is now routinely used in the setting of MIP. Different criteria for measurement and interpretation of IOPTH were suggested. One study compared different criteria (Halle, Miami, Vienna, and Rome) and noted greater accuracy when the Miami criterion was used. This criterion is defined as a PTH drop of 50% or more from the highest of either preoperative or pre-excision baseline value to the level at 10 minutes after hyperfunctioning parathyroid gland excision indicates surgical cure ^54^. Richards *et al.* suggested, as a surgical cure criterion, a 50% fall into the normal range and showed a decrease in the rate of surgical failure, especially in patients with multigland disease ^55^. The most recent guideline indicates use of the latter criterion ^48^. Rarely (in well under 5% of cases), a patient who meets these intraoperative PTH criteria for “cure” will not be cured.

Improvement in various organs affected by PHPT is noted after curative parathyroidectomy. Studies show beneficial effects in bone disease, including improvement in BMD, bone microstructure, and decrease in bone markers ^56– 58^. A reduction in the recurrence rate of nephrolithiasis has also been noted ^59, 60^. Tassone *et al.* recently evaluated the renal function of 109 patients before and after surgery and noted that surgical cure halts the deterioration of renal function in patients with PHPT ^61^. Other studies suggest that curative surgery improves quality of life ^43, 46, 47, 62^.

## Medical management

Many patients do not meet surgical criteria and elect not to pursue parathyroid surgery. There are also patients who meet surgical criteria but who opt for a non-surgical approach. There are still other patients for whom medical contraindications preclude a surgical approach ^1^.

### Vitamin D and calcium

Calcium intake should follow the guidelines established for the general population. There is no reason for calcium restriction in patients with PHPT ^48^. In patients with 25-hydroxyvitamin D levels in the insufficient or frankly deficient range (namely, less than 30 or 20 ng/mL, respectively), vitamin D should be given cautiously. The amount of vitamin D can be titrated from 400 to 800 IU/day as the situation warrants. Many experts feel that in PHPT, a desirable goal is for the 25-hydroxyvitamin D to be maintained above 30 ng/mL ^48, 63^.

### Bisphosphonates

Misiorowski evaluated the effect of a 2-year course of alendronate in symptomatic patients who refused surgery ^64^. The lumbar spine and femoral neck sites improved. Khan *et al.* evaluated patients with asymptomatic PHPT in an experimental design that called for 2 years of continuous therapy compared with a group receiving placebo in the first year, followed by a crossover to alendronate in year 2 ^65^. Lumbar spine and hip sites improved along with a reduction in bone markers. There was no improvement in BMD at the distal radius. The group that was crossed over from placebo to alendronate in year 2 showed a slope of improvement in BMD at the lumbar spine that mirrored the increase in BMD in year 1 in the group that received alendronate from the onset of the study.

Bisphosphonates have also been studied in NPHPT. This study matched groups receiving cholecalciferol with or without alendronate for 1 year. Similar to the study by Khan *et al.* with hypercalcemic PHPT, lumbar spine and hip BMD improved along with a reduction in bone turnover markers ^66^. In virtually all studies, serum calcium, PTH, and urinary calcium excretion did not change ^64– 66^.

### Calcimimetic therapy

The calcimimetic, cinacalcet, has shown utility in PHPT. In general, cinacalcet normalizes serum calcium and modestly reduces PTH ^67– 69^. These studies have not shown any positive effects of cinacalcet on BMD or non-specific symptoms of the disease ^68, 69^. Peacock *et al.* evaluated patients with severe or mild disease and found normalization in serum calcium and decrease in PTH in both groups ^69^. Faggiano *et al.* compared a group of patients receiving alendronate plus cinacalcet and another group receiving cinacalcet only ^70^. In both groups, there was normalization of calcium and decrease in PTH; however, there was a significant improvement in BMD only in the group using alendronate.

These experiences give guidance as to which pharmacological agent one would select in those who are candidates for drug therapy. A cautionary note is that many patients with asymptomatic PHPT do not have to be considered for pharmacological therapy because their BMD is not low and their serum calcium is within 1 mg/dL of the upper normal range. In these subjects, there is no need to consider pharmacological therapy. For those whose bone density is low, however, a bisphosphonate would be a logical choice. For those whose serum calcium is in the range in which reduction is a desired goal, cinacalcet would be the logical choice. For those who present with both low BMD and serum calcium of greater than 1 mg/dL above the normal range, combination therapy would make sense ^71^.

## Conclusions

PHPT is one of the most common endocrine diseases. Recent advances in the presentations of PHPT, with regard to both the hypercalcemic and normocalcemic forms, have led to new concepts regarding management. The revised guidelines reflect a more proactive approach to evaluation and management. We are still in need of more information regarding NPHPT as well as putative off-target manifestations of PHPT. For patients who have not met any guidelines for surgery and are not going to have surgery, conservative management with or without pharmacological intervention is an option.
